# Association of Vitamin B12 Deficiency with Fatigue and Depression after Lacunar Stroke

**DOI:** 10.1371/journal.pone.0030519

**Published:** 2012-01-20

**Authors:** Marjolein Huijts, Annelien Duits, Julie Staals, Robert J. van Oostenbrugge

**Affiliations:** 1 Department of Neurology, Maastricht University Medical Centre, Maastricht, The Netherlands; 2 Department of Psychiatry and Psychology, Maastricht University Medical Centre, Maastricht, The Netherlands; 3 School for Mental Health and Neuroscience (MHeNS), Maastricht University, Maastricht, The Netherlands; 4 Cardiovascular Research Institute Maastricht (CARIM), Maastricht University, Maastricht, The Netherlands; Cardiovascular Research Institute Maastricht, Maastricht University, Netherlands

## Abstract

**Background:**

In lacunar stroke patients vitamin B12 deficiency is often found and a relationship with the degree of periventricular white matter lesions (pWMLs) is suggested. Given the known relationships between WMLs and depression and between depression and fatigue after stroke, we studied both depression and fatigue in lacunar stroke patients with and without vitamin B12 deficiency.

**Methods:**

In 40 first-ever lacunar stroke patients vitamin B12 levels were determined and self-report questionnaires for fatigue and depression were completed three months after stroke.

**Results:**

Lacunar stroke patients with vitamin B12 deficiency (N = 13) reported significantly more fatigue (90.7 versus 59.4; *p* = .001) and depressive symptoms (6.62 versus 3.89; *p*<.05) than those without (N = 27). In regression analyses, vitamin B12 deficiency was significantly and independently associated with the presence of severe fatigue and clinically significant depression.

**Conclusions:**

Our preliminary results suggest a relationship between vitamin B12 deficiency and increased levels of fatigue and depression in lacunar stroke patients. If these findings could be replicated in a larger and general stroke sample, this would open treatment options and may improve quality of life after stroke.

## Introduction

Recently, we found that around 30% of lacunar stroke patients were vitamin B12 deficient [1] while in the normal elderly population this is only 10–15% [2]. This deficiency was related to the degree of periventricular white matter lesions (pWMLs) [1]. WMLs in turn are known to be related to depression [3], while post-stroke depressive symptoms have been associated with an increased risk of post-stroke fatigue [4]. Recently, the prevalence of depression in lacunar stroke patients was found to be 35% [5]. The prevalence of fatigue in these patients is 38% [6]. So far, there are no data available on the relationship between vitamin B12 deficiency and either depression or fatigue in lacunar stroke patients. In the present study we aimed to compare levels of fatigue and depression in first-ever lacunar stroke patients with and without vitamin B12 deficiency.

## Methods

### 1. Study population

We consecutively included 40 first-ever lacunar stroke patients presenting at the Neurology Department of the Maastricht University Medical Centre between February 2009 and October 2010. Lacunar stroke was defined as an acute stroke syndrome with an ischemic lesion on brain MR compatible with the occlusion of a single perforating small artery (subcortical, demarcated, and a diameter <20 mm). Patients with severe co-morbidity, either neurological or psychiatric, were excluded. Patients were assessed at 3 months after stroke to exclude acute phase effects.

### 2. Ethics statement

The study was approved by the Medical Ethical Committee of the Maastricht University Medical Centre and all patients signed informed consent.

### 3. Assessments

Fatigue was measured by self-report with the Checklist Individual Strength (CIS) [7]. The questionnaire contains 20 statements with total scores ranging from 20 to 140. The patient has to indicate on a 7-point scale to what extent the statement is applicable for him/her. A score >76 indicates severe fatigue [7].

Symptoms of depression were measured by self-report with the Hospital Anxiety and Depression Scale-Depression subscale (HADS-D) [8]. This subscale consists of 7 items with 4 possible statements relating to the emotional aspects of depression. Total scores range from 0 to 21. This subscale does not include physical and cognitive symptoms, including fatigue. As such, it is suitable to use in somatic populations. Since suicidal items are lacking, the instrument is less appropriate to assess the severity of depression. However, a score >10 is considered clinically significant.

Stroke severity was measured with the National Institutes of Health Stroke Scale (NIHSS) [9] on admission.

### 4. Vitamin B12 level

Vitamin B12 was measured in serum using a solid-phase time-resolved fluoroimmunoassay. Vitamin B12 level <150 pmol/L was considered deficient [1], since this is clinically as well as scientifically used as a cut-off to decide for vitamin B12 deficiency [10]. Also, Pieters et al [1] used a cut-off of 2.5% at both ends, which resulted in reference values between 150 and 630 pmol/L. To further substantiate this lower reference value, they applied the Bhattacharya-technique and found the lower limit also to be at 150 pmol/L.

### 5. Brain MRI

On brain MRI (standard T2-weighted and FLAIR sequences), two experienced neurologists (JS and RvO) individually graded pWMLs based on the Fazekas scale [11]. Extensive white matter lesions were defined according to Fazekas classification as T-2 weighted irregular periventricular hyperintensities extending into the deep white matter. In case of disagreement, lesions were ascertained by consensus.

### 6. Statistical analysis

We used PASW Statistics 18 software. Differences between groups were analyzed with Mann-Whitney U-tests and Pearson Chi-square. We used logistic regression analyses to test associations between the presence of severe fatigue and clinically significant depression, and vitamin B12 status, adjusted for the presence of extensive pWMLs. Since depression is found to be related to low vitamin B12 levels in the elderly population [12], we also adjusted for age.

## Results

We included 40 lacunar stroke patients with a mean age of 67.9 years (SD = 12.6), of which 15 (38%) females. Mean NIHSS score was 3.42 (SD = 2.30). All patients completed the questionnaires. Vitamin B12 deficiency was present in 13 (33%) patients. The mean CIS score was 69.6 (SD = 27.6) and the mean HADS-D score was 4.7 (SD = 3.9). Severe fatigue was reported by 17 (42.5%) patients and clinically significant depressive symptoms by 6 (15%) patients.

Table 1 presents the results of the fatigue and depression assessments in patients with and without vitamin B12 deficiency. Patients with vitamin B12 deficiency had significantly higher scores of overall fatigue and depressive symptoms.

**Table 1:** Demographics and mean scores for lacunar stroke patients with and without vitamin B12 deficiency.

	With vitamin B12 deficiency (*N* = 13)	Without vitamin B12 deficiency (*N* = 27)	*p*
Age (SD)	66.6 (14.0)	68.6 (12.1)	.795
Female (%)	7 (53.8)	8 (29.6)	.138
NIHSS score (SD)*	3.0 (2.0)	3.6 (2.5)	.506
CIS score (SD)	90.7 (20.0)	59.4 (25.0)	**.001**
Severe fatigue (%)	11 (84.6)	6 (22.2)	**.000**
HADS-D score (SD)	6.6 (4.0)	3.7 (3.5)	**.019**
Clinically significant depressive symptoms	4 (30.8)	2 (7.4)	.053

*NIHSS scores were only available for 9 (with vitamin B12 deficiency) and 15 (without deficiency) patients.

Logistic regression analyses revealed that vitamin B12 deficiency was significantly associated with the presence of severe fatigue (*p* = .002), independent of age and the presence of extensive pWMLs (table 2).

**Table 2:** Logistic regression analyses on the presence of severe fatigue.

Model	Independent variables	β	SE	OR	95% CI for OR	p-value
1	B12 deficiency	2.958	.897	19.250	3.316–111.747	**.001**
2	B12 deficiency	3.043	.971	20.976	3.130–140.549	**.002**
	Age	−.039	.037	.961	.895–1.033	.280
	Severe pWMLs	−.038	1.017	.963	.131–7.068	.970

Model 1: univariate analysis. Model 2: multivariate analysis with adjustments for age and the presence of severe pWMLs.

## Discussion

The results showed vitamin B12 deficiency in 33% of our sample of lacunar stroke patients. Those with deficiency reported more fatigue and depressive symptoms than those without. In addition, vitamin B12 deficiency was found to be associated with fatigue and depressive symptoms independent of age and the presence of pWMLs.

Depression and fatigue are known to be strongly correlated [13]. Besides, fatigue is a common symptom of depression. To increase the construct validity, we used the HADS-D scale for measuring depressive symptoms. This scale does not contain somatic items including fatigue, and as such we reduced the overlap between the assessments of depression and fatigue. Limitation of this study however is the cross-sectional design, which makes it impossible to infer causality. To make causal inferences from the relationships found, we need longitudinal studies but preferably a randomized design.

Although we found a relationship between vitamin B12 deficiency and pWMLs in earlier work [1], WMLs did not affect the relationship between vitamin B12 deficiency and either fatigue or depressive symptoms. In this study we did not intend to examine the direct relationships between pWMLs and both fatigue and depression on the other hand. Larger samples are needed to study all interrelationships. So far, the present results, though preliminary, do not suggest a mediating role for WMLs in explaining the relationship between vitamin B12 deficiency and both fatigue and depression. Vitamin B12-induced anemia, on the other hand, could be a mediating factor, since this may induce fatigue. However, based on a retrospective search on hemoglobin-values, there was only one patient with values below threshold.

Although not significant in our sample, prevalence rates of vitamin B12 deficiency in general populations are higher for women than for men [2]. It was suggested that this difference was related to hormone replacement therapy, but this appeared not to be a significant contributor to low vitamin B12 status [14].

Data about stroke severity (NIHSS) were not available for all patients. Although we cannot exclude that stroke severity influences the degree of fatigue and depression, we do not expect a major effect as, in general, lacunar stroke patients present with relatively mild neurological deficits.

Given the small sample size and cross-sectional design, our findings are statistically only preliminary. However, since vitamin B12 deficiency is easily treatable by suppletion, the results are clinically relevant making further research worthwhile. Both depression and fatigue may have a negative impact on rehabilitation and quality of life after stroke [15]. If these findings could be replicated in a larger and general stroke sample, this would open treatment options and may improve quality of life after stroke. Therefore, we suggest further research in these populations as well as in elderly people with vitamin B12 deficiency. We also suggest adding functional assessments of vitamin B12 deficiency such as homocysteine and methylmalonic acid measurements. To infer causality, intervention studies with vitamin B12 suppletion are warranted.
